# Severe Cerebral *Falciparum* Malaria with Dengue Coinfection: A Case Report

**Published:** 2018

**Authors:** Yunsheng ZHAO, Xia WU, Fei LIAO

**Affiliations:** 1. Dept. of Clinical Laboratory, The First Hospital of Qinhuangdao, Qinhuangdao, Hebei Province, China; 2. College of Laboratory Medicine, Chongqing Medical University, Chongqing, China

**Keywords:** *Falciparum* malaria, Dengue, Coinfection, Prognosis

## Abstract

A rare severe case of the coinfection of cerebral *falciparum* malaria and dengue is reported in this paper. The patient was a 50 yr old male in the north of China, who returned from Uganda not long ago and suffered unconsciousness after fever for 4 d before the *falciparum* malaria and dengue infection was diagnosed via blood smear microscopy, RT-PCR and serology, although the antimalarial and symptomatic treatment was carried out, ultimately the patients died of septic shock because of multiple organ dysfunctions. This case report showed such a coinfection was prone to cause severe acute consequence. The timely diagnosis and delicate treatment are crucial for prognosis of patients.

## Introduction

Malaria and dengue are common insect-borne infectious pathogen, which is epidemic in tropical and subtropical regions of Asia, America, and Africa and spread by mosquito bites (1, 2). However, the cases about coinfection of those two pathogens were reported rarely since 2005 (3, 4). Malaria and dengue were not prevalent in northern China in recent years, but with the increasing of personal mobility, the imported infection by malaria and dengue increased gradually (5, 6). *Falciparum* malaria has the highest incidence and mortality rate in malaria infection, in which severe cerebral *falciparum* malaria is rare with the incidence of about 2% and the mortality of 9%∼31%; such an infection was characterized by pernicious attack, rapid development and short duration (7).

Here, a rare case of imported severe cerebral *falciparum* malaria with dengue coinfection in north China is reported.

## Case report

A 50-yr-old man in the north of China went to Uganda to search for a work on Jul 14, 2016, and returned home on Jul 25. No obvious cause of fever with a temperature of 38.5 °C and occasional cough were found on Jul 30. After intravenous cephalosporin medication in local clinic for 3 d since the night of July 30, his body temperature dropped to normal. The patient became partially unconscious at 18:00 on Aug 2, and was sent to the Second Hospital of Changli County firstly, and then transferred to our hospital due to dangerous condition. Upon admission, the patient was found to have body temperature of 38.5 °C, unconsciousness, irritability, BP of 137/93 mm Hg and heart rate of 122 times/min with the consideration of febrile diseases. After being given intravenous cefoperazone sulbactam symptomatic treatment in the fever clinic, his status showed no improvement and the patient was transferred to intensive care unit for further treatment at 10:30 on Aug 3.

In IUC, physical examination gave the following results as blurred consciousness, irritability, high blood pressure, skin and sclera yellow dye, but few of other positive changes. The patient was treated with sedative, acid suppression, liver protection, clearing mind and anti-infection of meropenem. Considering the *falciparum* malaria with the warning of local CDC, the patient was transferred to the Third Hospital of Qinhuangdao (the infectious diseases hospital of Qinhuangdao) for continued treatment at 17:00. Considering the patient with cerebral *falciparum* malaria associated with liver and myocardial damage, more tests were performed and results indicated a lung infection and metabolic acidosis.

The patient was given sodium bicarbonate intravenous drip to correct acidosis, magnesium isoglycyrrhizinate, and reduced glutathione to protect liver, pantoprazole intravenous drip to protect the gastric mucosa, meropenem to anti-infection, diazepam and cockstailytic for sedation treatment, artemether and dihydroartemisinin-piperaquine tablets for anti-malaria treatment. Unfortunately, the patient’s status showed no improvement till 00:20 on Aug 4, and was transferred to ICU of Beijing Ditan Hospital for further treatment at 04:47 on Aug 4. After antimalarial therapy with intramuscular artemether, anti-infection treatment with intravenous cefmetazole and other symptomatic treatment, the disease did not relieve and acidosis became heavier; the patient had already in a deep coma at 15:00 on Aug 5, and then died of septic shock at 20:40.

The study was approved by the hospital.

### Laboratory examination

#### 1. The Results of initial investigation

Blood sample showed hemoglobin of 127 g/l, red blood cell count of 4.29×10^12^/l, total white blood cell count of 6.71×10^9^/l, 85.5% neutrophil and platelet count of 14×10^9^/l; his liver function test showed hyperbilirubinaemia of 247.68 μmol/l and transaminitis with serum ALT 84.4 U/l, AST 113.4 U/l, LDH 492 U/l, total bile acid 32.46 μmol/l, rglutamyltranspeptidase 431.2 U/l, blood ammonia 35.8 μmol/l and the total protein 62.3 g/l; his serum D- dimmer, fibrinogen and procalcitonin raised to 13.72 μg/ml, 5.38 g/l and 6.23 ng/ml, respectively; bicarbonate was 16.7 mmol/l and plasma protamine paracoagulation was weakly-positive; urine analysis revealed elevated bilirubin, protein, occult blood and ketone.

#### 2. The microscopic examination of peripheral blood smears

**Fig. 1:**
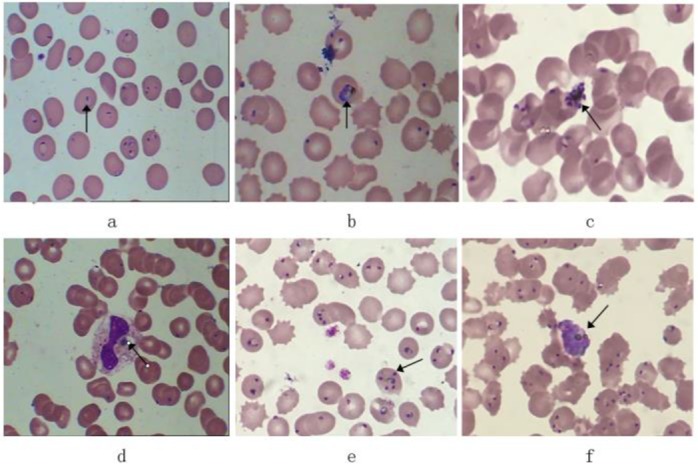
The microscopic examinations of peripheral blood smear of *Plasmodium falciparum* infection (Wright’s staining, ×1000) Notes: a. early trophozoites (our hospital); b. big trophozoite; c. schizonts; d. malaria pigment; e. early trophozoites (the Third Hospital); f. mixed metabolites

Red blood cell (RBC) morphology was normal and about 40% of them contained 1–2 mainly or occasionally 3 early trophozoites of *Plasmodium falciparum*. The early trophozoites were annular and some were located on the edge of RBC, the ring was slender and it accounted for 1/4–1/5 of the diameter of RBC, the ring contained 1–2 nucleuses, which was dark purple, dense, round or oval. Big trophozoite was visible and occasionally schizonts in the smear. The malaria pigment phagocytosed by neutrophils or mononuclear cells was visible occasionally; it showed granular clumps of dark brown or yellowish brown. Finally, infection by *P. falciparum* was identified. Blood smear examination was performed again at the Third Hospital of Qinhuangdao after 4 h interval, the result showed that approximately 70% of RBC containing early trophozoites, in which three parasites were easy to be seen and 7 rings occasionally, furthermore mixed metabolites of *Plasmodium* were visible easily (Fig. 1).

#### 3. The reticulocyte (RET) scatter plot by Sysmex XE-2100 hematology analyzer

**Fig. 2:**
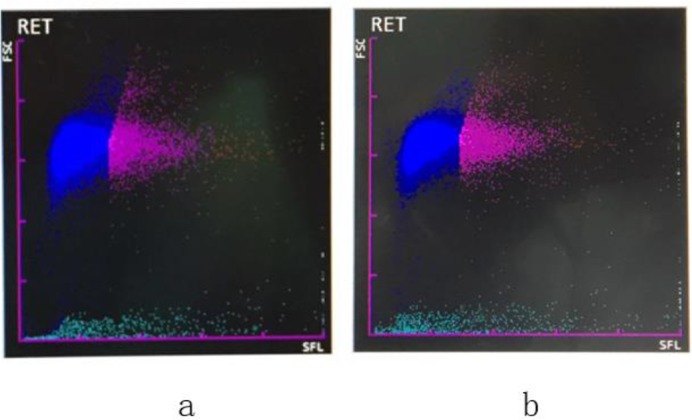
The RET scatter plot of *Plasmodium falciparum* infection Notes: forward scatter (FSC); side fluorescence (SFL); a. the result of our hospital; b. the result of the Third Hospital of Qinhuangdao. The blue plot represented erythrocyte, pink plot represented reticulocyte.

Take the patient’s blood samples from our hospital and the Third Hospital of Qinhuang-dao to analyze the RET scatter plots respectively, the results showed RET increased and the percentage of RET to RBC were 5.8% and 9.3% respectively (Fig. 2).

#### 4. The diagnosis of CDC

In the Qinhuangdao CDC, the serological detection result supported the mixed infection of *P. falciparum* by the colloidal gold method. In the Hebei Provincial CDC, dengue real-time polymerase chain reaction (RT-PCR) was positive, serum IgM was positive but IgG against the virus was negative by ELISA.

## Discussion

Malaria and dengue are widely infective among individuals in tropical and subtropical regions via mosquito bites, but they are not epidemic in northern China, especially a case of imported severe cerebral *falciparum* malaria with dengue coinfection is rare. The early clinical symptom of this patient was untypical. The limitation on the basic health technology condition for the corresponding auxiliary examination led to unclear diagnosis at early stage of disease and delayed the timely treatment till the development of severe cerebral *falciparum* malaria. His main symptoms included fever and coma, besides multiple organ function damage. Together with the “Uganda” business history, the patient was confirmed by the coinfection of *falciparum* malaria and dengue after the peripheral blood smears and serological examination but eventually died of septic shock due to the severe conditions. The progression of the disease was only a week from the onset to death. Through the comparison of blood smear examination results showed the rapid reproduction of *P. falciparum* in vivo, there appeared a high parasitemia about 70% of RBC containing malarial early trophozoites at later stage, and big trophozoite and schizont of *P. falciparum* can be seen in peripheral blood; these laboratory examinations indicated a poor prognosis (8).

The main cause of dangerous condition of this patient was the coinfection of *Plasmodium* and dengue virus, showing aggravation of clinical symptoms and complexity of the condition (9). In this case, PLT decreased obviously, but the hemoglobin did not decrease obviously; these changes were different from the common symptoms of *falciparum* malaria, probably due to the coinfection of dengue. Namely, the coinfection of *Plasmodium* and dengue increased the complexity of laboratory diagnosis and it probably led to delay the treatment for the prevention of organ dysfunction.

The gold standard for the diagnosis of malaria is the finding of *Plasmodium* in the smear of peripheral blood or bone marrow (10). The patient had about 40% of RBC containing *falciparum* malarial ring forms besides the clinical manifestations of fever and coma, leading to the diagnosis of cerebral *falciparum* malaria. The pathological process of *falciparum* malaria consumed a large amount of PLT (11), and the laboratory results also showed the plasma protamine paracoagulation was weakly-positive and PLT decreased significantly. *Plasmodium* propagated rapidly in vivo in 4 h, the red blood cells containing *Plasmodium* increased from 40% to 70% and the number of early trophozoites increased from mainly one or two to mainly three or even more. The RET scatter plot showed significant cell group and the percentage of reticulocytes increased from 5.8% to 9.3%, which may be the blood analyzer mistaked the erythrocyte that infected by *Plasmodium* as reticulocytes and indicated RET scatter plot can be used for the screening of *Plasmodium* infection. Using abnormal scatter plot can screen *Plasmodium* quickly and easily (12).

Therefore, in daily work, we should pay attention to the scatter plot of blood analysis to confirm any abnormal changes and make the necessary blood smear according to the prompt information, for the timely diagnosis of the coinfection and prevent misdiagnosis.

## Conclusion

The case about coinfection of severe cerebral *falciparum* malaria with dengue is rare, the timely diagnosis and delicate treatment are crucial for prognosis of patients.
